# Drug/Lead Compound Hydroxymethylation as a Simple Approach to Enhance Pharmacodynamic and Pharmacokinetic Properties

**DOI:** 10.3389/fchem.2021.734983

**Published:** 2022-02-14

**Authors:** Soraya S. Santos, Rodrigo V. Gonzaga, Cauê B. Scarim, Jeanine Giarolla, Marina C. Primi, Chung M. Chin, Elizabeth I. Ferreira

**Affiliations:** ^1^ Laboratório de Planejamento e Síntese de Quimioterápicos Potencialmente Ativos Em Doenças Negligenciadas (LAPEN), Departamento de Farmácia, Faculdade de Ciências Farmacêuticas, Universidade de São Paulo – USP, São Paulo, Brazil; ^2^ Laboratório de Pesquisa e Desenvolvimento de Fármacos (LAPDESF), Departamento de Fármacos e Medicamentos, Faculdade de Ciências Farmacêuticas, Universidade Estadual de São Paulo “Júlio de Mesquita Filho” (UNESP), Araraquara, Brazil; ^3^ The Scripps Research Institute, Jupiter, FL, United States; ^4^ Centro de Pesquisa Avançada Em Medicina (CEPAM), Faculdade de Medicina, União Das Faculdades Dos Grande Lagos (UNILAGO), São José Do Rio Preto, Brazil

**Keywords:** hydroxymethylation, hydroxymethyl compounds, hydroxymethyl drug, prodrug design, hydroxymethylnitrofurazone

## Abstract

Hydroxymethylation is a simple chemical reaction, in which the introduction of the hydroxymethyl group can lead to physical–chemical property changes and offer several therapeutic advantages, contributing to the improved biological activity of drugs. There are many examples in the literature of the pharmaceutical, pharmacokinetic, and pharmacodynamic benefits, which the hydroxymethyl group can confer to drugs, prodrugs, drug metabolites, and other therapeutic compounds. It is worth noting that this group can enhance the drug’s interaction with the active site, and it can be employed as an intermediary in synthesizing other therapeutic agents. In addition, the hydroxymethyl derivative can result in more active compounds than the parent drug as well as increase the water solubility of poorly soluble drugs. Taking this into consideration, this review aims to discuss different applications of hydroxymethyl derived from biological agents and its influence on the pharmacological effects of drugs, prodrugs, active metabolites, and compounds of natural origin. Finally, we report a successful compound synthesized by our research group and used for the treatment of neglected diseases, which is created from the hydroxymethylation of its parent drug.

## Introduction

Hydroxymethylation is, in general, a simple chemical reaction that allows the addition of the -CH_2_OH group to some starting materials, such as alkanes and aromatic acidic compounds, which is carried out by employing normally aqueous formaldehyde (37–41%) in a basic medium**.** In addition, there are some hydroxymethylation reactions that use metal catalysts and paraformaldehyde (Lia and Wu, 2015) and others employing an interesting quadruple relay catalyst (Tang et al., 2020).

Hydroxymethylation is also reported in biosynthetic pathways, for example, in the biosynthesis of plant secondary metabolites, as observed in alkaloids and coumarins. Often, the introduction of the hydroxymethyl group aims to obtain more hydrophilic derivatives than the parent compound. Moreover, the hydroxymethyl function allows the molecular recognition by bioreceptors through the establishment of hydrophilic and hydrophobic interactions with the target, acting as a pharmacophore. Hydroxymethyl derivatives have biological importance, as observed in vitamins, for example, vitamin B6 and nucleosides, the latter of which have been employed as prototypes for antivirals available in therapy. There are examples of hydroxymethyl drugs with higher metabolic stability and target interaction, with diverse clinical indications, such as antihypertensives and anticancer agents, which include nucleoside, non-nucleoside, and natural product-derivatives. In nucleoside analogs, the hydroxymethyl group plays a role in the activation of these agents, as it undergoes several phosphorylation steps to be activated (Huennekens et al., 1959; Bundgaard, 1985b).

There are various hydroxymethyl bioactive compounds, of which some are used in therapeutics, while others are under research. Besides this, hydroxymethylation is often used to design prodrugs. According to Zawilska et al. (2013), almost 10% of the drugs introduced in therapeutics are considered prodrugs. The term “prodrug” refers to reversible derivatives of the drugs, which *in vivo*, by chemical or enzymatic reaction, release the active compound. This promising molecular modification can improve the physicochemical, biopharmaceutical, pharmacokinetic, and pharmacodynamic properties of the prototype (Chung and Ferreira, 1999; Chung et al., 2005; Silva et al., 2005; Chung et al., 2008; Rautio et al., 2008; Huttunen et al., 2011; Zawilska et al., 2013; Delplace, Couvreurc, Nicolas, 2014). Hydroxymethyl derivatives could be quite useful as a prodrug as well as acting as a key intermediate for obtaining prodrugs, for instance, to improve drug solubility, bioavailability, change the route of administration (Bansal et al., 1981; Varia and Stella, 1984a; Varia and Stella, 1984b; Varia et al., 1984), reduce toxicity and increase bioavailability (Mahfouz et al., 1999), and design mutual prodrugs (Scriba, 1996).

Herein, we will not only explore the synthetic approaches for obtaining the hydroxymethylated compounds but also the biological aspects of hydroxymethylation resulting in prodrugs and drug and bioactive compounds under research. In addition, we mention a successful hydroxymethylation obtained by our research group from a hydroxymethylated synthesis intermediary, named hydroxymethylnitrofurazone (NFOH). NFOH showed higher antichagasic activity than the parent compound (nitrofurazone) and the current drug of choice for Chagas disease treatment (benznidazole).

## Prodrug Design Approaches: Effects of Hydroxymethylation on the Pharmacokinetic Properties of Drug/Bioactive Compounds

### Improvement of Solubility and Bioavailability

Solubility has been a great challenge for drugs/bioactive compounds to be introduced as therapeutics (Sanches and Ferreira, 2019). Around 40% of the 200 top drugs already in the pharmaceutical market have a problem with solubility (Takagi et al., 2006), and about 70% of compounds in the pipeline are practically insoluble in water (Censi and Martino, 2015). This barrier can compromise the translational phase, which means the passage from lead to drug candidate, which is one of the problems in the “valley of death” (Basavaraj and Betageri, 2014; Gamo, et al., 2017). Formulation approaches can be used for overcoming this problem, but the prodrug design is also a process that has been successfully used with either drugs or bioactive compounds.

Some representative examples are given as follows: the contribution of Hans Bundgaard in this objective was outstanding in the 80′s (Bundgaard, 1985a). Bundgaard published more than 300 articles before his death in the 90`s, most of them about the prodrug design, especially focused on improving solubility. It is worth noting that *N*-hydroxymethyl intermediates (Bundgaard, 1985b) were explored by Bundgaard, and they can be used as key molecules to obtain prodrugs with better solubility, such as acyloxymethyl esters (Bundgaard and Falch, 1985).

Hydroxymethylnitrofurantoin (**1**, URFADYN®) is a nitrofurantoin (**2**, Furadantina MC®) prodrug, and both the parent drug and prodrug are used to treat urinary tract infections (Sorel and Roseboom, 1979; Bundgaard and Johansen, 1980a). The hydroxymethylnitrofurantoin metabolism and excretion in healthy volunteers revealed that it is quickly converted to nitrofurantoin (Supplementary Scheme 1S). In addition, Bundgaard and Johansen (1980b) evaluated the mechanism of decomposition of the *N*3-hydroxymethyl prodrugs created from hydantoins, such as phenytoin, nitrofurantoin, and 5,5-dimethylhydantoin. Fast conversion was observed at pH 7.4 and 37°C (half-life ranges from 0.1 to 6.9 s). In addition, they had higher water solubility than the parent drug, as for example, phenytoin showed 0.032 mg/ml of solubility and its derivative 3-(hydroxymethyl)phenytoin presented 0.14 mg/ml (Bundgaard, and Johansen, 1980a). The acid stability of prodrugs was another important feature, which allowed their complete cleavage only in a neutral environment.

Prodrugs of theophylline (**3–6**) were obtained from the acylation of 7-(hydroxymethyl)theophylline (**7**) and the alkylation of theophylline (**8**) with an acyloxymethyl halide (Sloan, and Bodor, 1982) (Supplementary Scheme 2S). Considering the diffusion of drug and respective prodrugs through the hairless mouse skin assay, 7-(hydroxymethyl)theophylline (**7**) was able to carry theophylline topically, being almost 5-fold more effective than the prototype. Moreover, there was a complete conversion of the prodrugs 7-(hydroxymethyl)theophylline (**7**) and 7-(butyryloxymethyl)theophylline (**3**) to their parent drug. On the other hand, 14.5 + 5% of the 7-(pivaloyloxymethyl)theophylline (**4**) prodrug remained intact at the end of the study. Kerr et al., (1998) also designed theophylline prodrugs from 7-alkylcarbonyloxymethyl derivatives, and they were evaluated in a topical delivery system. The most promising compound was 7-(hydroxymethyl)theophylline (**7**), which demonstrated lipid solubility similar to that of 7-(acetyloxymethyl)theophylline (**5**) and 7-(propionyloxymethyl)theophylline (**6**), though it was 10-fold more water soluble than theophylline (**8**). Additionally, this hydroxymethyl derivative showed *J*i (value of total theophylline species delivery) and Cs (total theophylline species retained in the skin) two times greater than its parent drug. Therefore, the prodrug showed greater carrier capacity and bioavailability than the active drug form.

1-(Hydroxymethyl)allopurinol (**9**) is the synthetic key intermediate for obtaining amino acid ester prodrugs of allopurinol (Bundgaard, 1985a; Bundgaard et al., 1990), in order to achieve the ideal water solubility for parenteral and/or rectal drug administration. Although it is not the compound responsible for biological activity, this intermediary has a remarkable importance in planning the series of latent forms in question. The prodrug degradation occurs in two steps. In the first step, the ester (**10**) is hydrolyzed, resulting in an unstable 1-hydroxymethyl intermediate (**9**) and then, in a fast non-enzymatic step, the parent drug is released (Supplementary Scheme 3S). This kind of kinetic decomposition was explored in an interesting article published by Bundgaard and Johansen (1980b). Table 1 exhibits the *N,N*-(diethylglycil) (**11**), *N,N*(*-*dipropylglycil) (**12**), and DL-(*N,N*-diethylalanyl) (**13**) allopurinol prodrugs designed by Bundgaard and Falch (1985) and their pharmacokinetic features.

**TABLE 1:** Log *p* and chemical and enzymatic stability of *N*-acyloxymethyl allopurinol prodrugs (**11–13**) (Bundgaard and Falch, 1985).
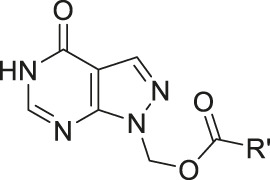

T(½) min					
**Compound**	**R′**	**Log^1^**	**pH 4.0**	**pH 7.4**	**80% human plasma**
**11**	*N,N*-diethylglycyl	0.20	28.9	49	10
**12**	*N,N*-dipropylglycyl	1.21	29.5	50	12
**13**	DL-*N,N*-diethylalanyl	0.72	32.5	21	17

1 Partition coefficients between *n-*octanol and 0.05 M borate buffer of pH 8.0 at 22 °C.

**SCHEME 1:**
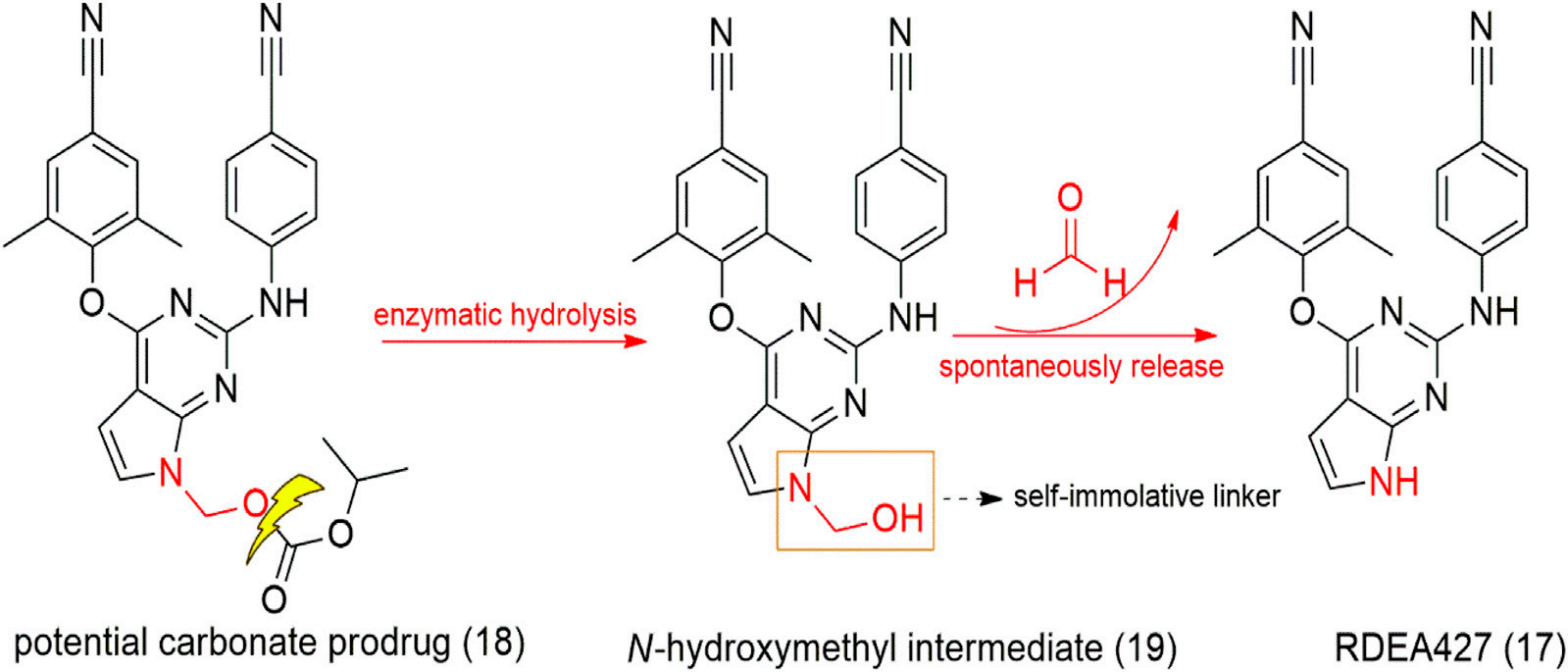
Design of the potential carbonate prodrug (**18**) and self-immolative release of RDEA427 (**17**).

The authors pointed out the design of compounds with better solubility in rectal administration, as well as an easily cleavable bond by enzymes. The prodrugs **(11–13**) showed log*P* values of 0.20, 1.21, and 0.72, respectively, which are better than those of allopurinol, which has log*P* of –0.55. Additionally, the half-lives (t½) in human plasma were 10, 12, and 17 min, respectively. These molecules were also relatively stable in pH 4.0 and 7.4, showing half-lives between 28.9 and 32.5 min at pH 4.0 and 21–50 min at pH 7.4 (Table 1). In order to improve allopurinol bioavailability and consider the prodrug degradation reported by Bundgaard and Falch (1985) (Supplementary Schemes 3S, 4S), the same research group designed new derivatives from the key intermediate 1-(hydroxymethyl)allopurinol, obtaining *N*-substituted [(3- or 4-aminomethyl)benzoyloxymethyl]allopurinol prodrugs (Bundgaard et al., 1990). The results were very promising, since compounds with good solubility and high chemical stability in a weakly acidic medium as well as high susceptibility to enzymatic hydrolysis in plasma were obtained. According to the authors, the prodrugs (**14–16**) (Scheme 1; Supplementary Scheme 4S) displayed higher absolute bioavailability after rectal administration (19, 38, and 41%, respectively) than allopurinol (3%). These compounds (**14–16**) were also much more lipophilic than the parent drug (–0.55) with log*P* values of 1.13, 1.12, and 0.97, respectively.

RDEA427 (17) is a new promising diarylpyrimidine derivative (DAPY-typed) non-nucleoside reverse transcriptase inhibitors (NNRTIs), which showed to be active against wild-type and a wide range of HIV-1 mutant strains (Huang et al., 2018). RDEA427 (**17**) reached the clinical trial stage, although it was discontinued due to inadequate properties. Many antiretroviral agents are prodrugs, such as tenofovir and raltegravir. In this context, the HIV-1 NNRTI drug candidate RDEA427 (**17**) was synthesized as a potential carbonate prodrug (**18**) from the *N*-hydroxylmethylated intermediate (**19**). Degradation of the prodrug occurs in two steps, as shown previously with the allopurinol prodrugs, via a self-immolative spacer degradation approach (Scheme 1). This prodrug was able to release the parent drug in human plasma, exhibiting an excellent activity against wild-type (EC_50_ = 0.0055 μM) and K103N/Y181C mutant HIV-1 strains (EC_50_ = 0.15 μM) and potent HIV-1 reverse transcriptase inhibitory activity (IC_50_ = 0.264 μM) (Huang et al., 2018; Sun et al., 2019) developed novel oral prodrugs from 5-fluorouracyl (5-FU) (**20**), the derivatives (**21–23**) (Scheme 3; Supplementary Scheme 5S). The hydroxymethylation is a crucial step for the synthesis of these prodrugs. The hydroxymethyl group was introduced at the *N*1-position of 5-FU (**24**), which was further conjugated to di-carboxylic acids, as monocarboxylate transporter 1 (MCT1)–targeting (**21–23**) (Scheme 3; Supplementary Scheme 5S). MCT1 is an important carrier derived from short-chain monocarboxylic acids that pass through the small intestine across the cell membrane, which could be exploited to increase oral absorption. These 5-FU prodrugs (**21–23**) were synthesized and exhibited the ability to release the active parent drug, presenting good chemical stability at different pH (pH 1.2, 6.8, and 7.4), in rat tissue homogenates and plasma. They displayed a half-life greater than 36 h, indicating great stability in the gastrointestinal tract. In addition, the authors showed that the prodrugs had a 13.1-fold higher permeability and 4.1-fold higher oral bioavailability than 5-FU, indicating excellent MCT1 targeting. Furthermore, in cytotoxicity assays in Caco-2 cells, all prodrugs exhibited better results than 5-FU (**20**), being 4.1- to 6.4-fold more potent than the free drug.

**SCHEME 2:**
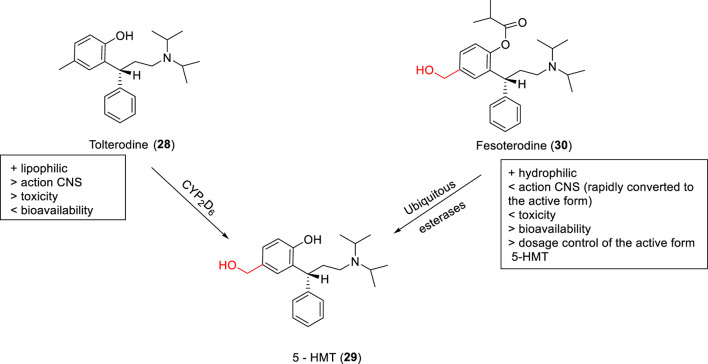
Tolterodine (**28**), prodrug fesoterodine (**30**), and metabolization to 5-HMT (**29**), the active derivative.

**SCHEME 3:**
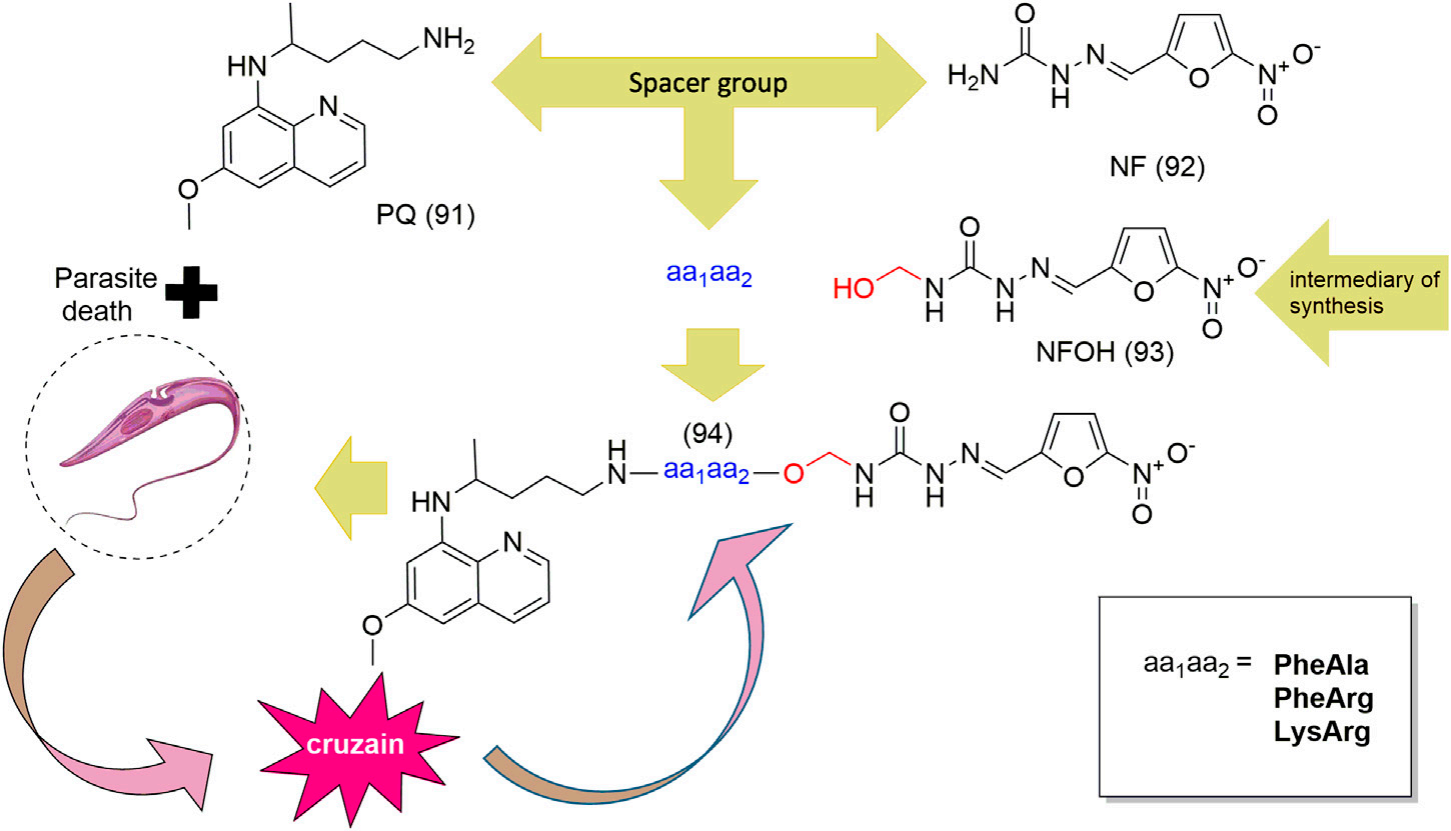
Design of mutual prodrugs and cleavage of cruzipain aa1aa2 dipeptide (**94**).

**FIGURE 1:**
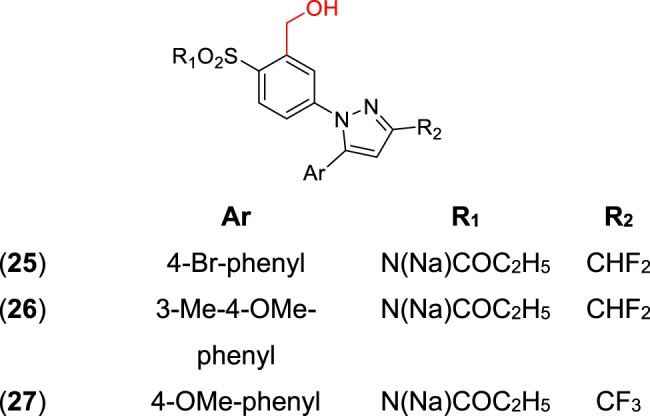
Hydroxymethyl and sulfamoyl prodrugs (**25**–**27**) are potentially anti-inflammatory.

Hydroxymethyl and sulfamoyl (SO_2_NH_2_) prodrugs were synthesized by Singh et al. (2006) as potential anti-inflammatory agents, acting as COX-2 inhibitors. In their first article, the best compounds of the series were the hydroxymethyl analogs (**25–27**) (Figure 1) with ED50 values of 5.6 and 6.3 mg/kg, and a percentage of COX- 2 inhibition of 14 and 13%. Additionally, an improvement of *in vivo* activity was observed, probably related to higher drug concentration released in the plasma. Another interesting feature verified was improved water solubility, which is important in parenteral administration. These prodrugs showed a 5–7-fold higher aqueous solubility (150–200 mg/ml at 25°C) than parecoxib sodium (22 mg/ml) and the celecoxib prodrug (15 mg/ml). Therefore, these prodrugs could help patients who feel severe pain, since a low volume injection would release high drug concentration upon biotransformation.

Tolterodine (**28**, Detrol®, Detrusitol®) is a competitive selective bladder M2 and M3 muscarinic receptor antagonist employed in the treatment of an overactive bladder. Tolterodine absorption occurs in the gastrointestinal tract and is metabolized to 5-hydroxymethyl tolterodine (5-HMT - active metabolite - **29**) by CYP2D6 enzymes present in the liver and intestine. However, due to genetic individuality, each person expresses a certain amount of this enzyme, which makes it challenging to use tolterodine in individuals with low expression of CYP2D6. In this context, Meese and Sparf (2012) designed a 5-HMT prodrug named fesoterodine (**30** – Scheme 2), with the aim of improving the drug’s bioavailability parameters. The prodrug has a hydroxymethyl group as the active metabolite, and fesoterodine showed lower blood–brain barrier permeation than tolterodine, which decreased toxicity in the central nervous system. Fesoterodine biotransformation to 5-HMT (29) is a fast and efficient step after oral administration performed by nonspecific esterase enzymes (Malhotra et al., 2009).

**SCHEME 4:**
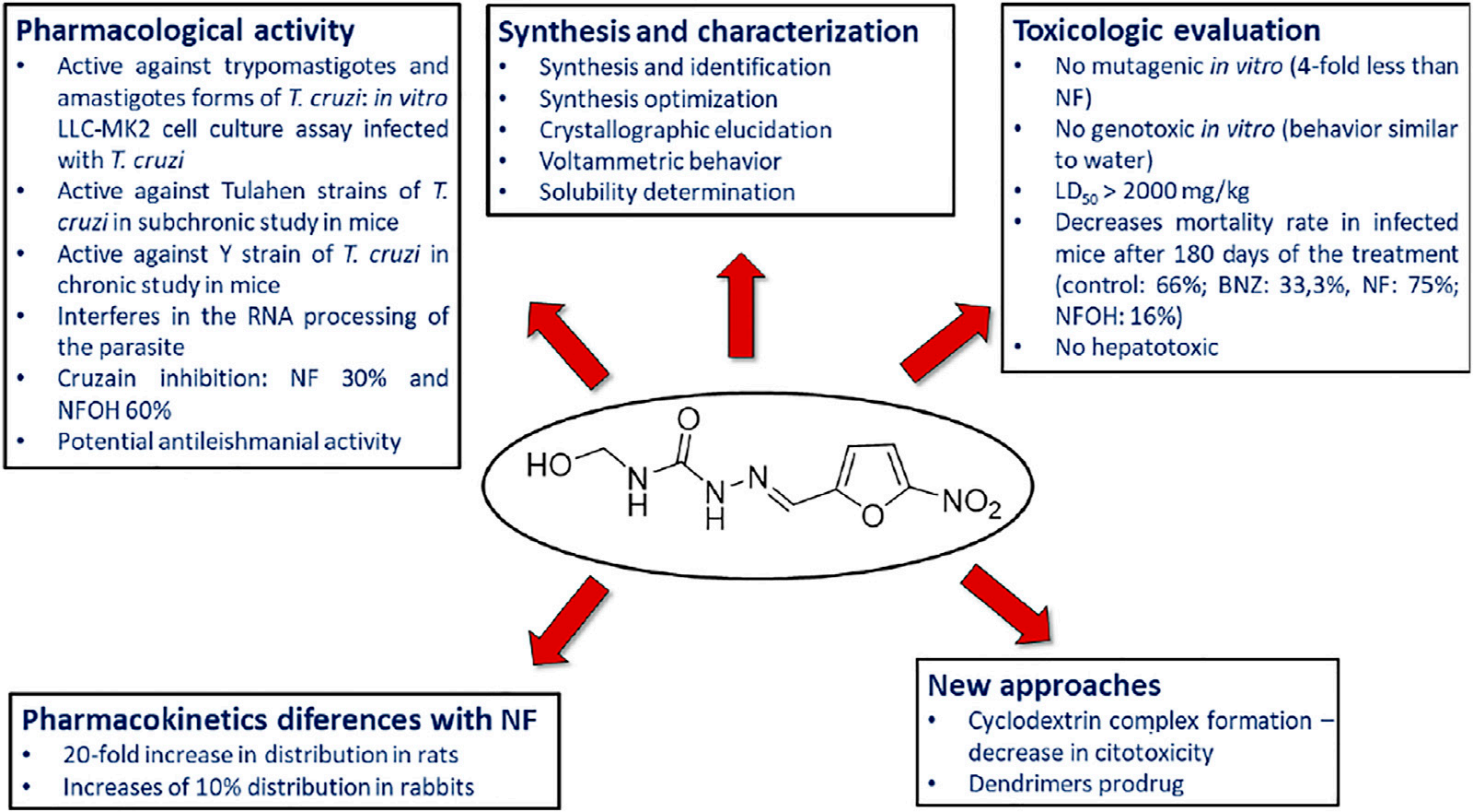
Summary of the results obtained from the evaluation of pharmacological activity, toxicity and other studies carried out with NFOH (**93**).

### Metabolic Stability and Influence of Metabolism on Activity

Friis and coworkers (1996) explored *N*-hydroxymethylated prodrugs of A-responsive carboxypeptidase. In their work, *N*-Z-protected dipeptides were *N*-hydroxymethylated at the *C*-terminal peptide bond. The stability assays in different pH solutions and two concentrations of carboxypeptidase-A demonstrated the importance of *N*-hydroxymethylation to the preservation of the structural integrity of the peptide. With respect to hydrolysis in the presence of carboxypeptidase A, the derivatives with CH_2_OH (**31**–**34**) were stable in the presence of the enzyme and showing small variation in half-life values (Table 2). Compound (**33**) without the *N*-hydroxymethylated group presented stability in pH solution; however, it suffered degradation in the presence of the enzyme. Therefore, the hydroxymethylated group provides higher enzymatic stability to these prodrugs.

**TABLE 2:** Chemical and enzymatic stability of prodrugs (**31–34**) (Friis et al., 1996).
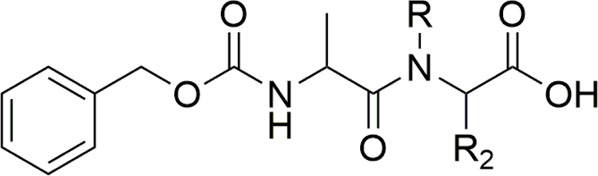

Compound	R2	R	pH 7.4 buffer^a^	Carboxypeptidase A (25 U/mL)^b^	Carboxypeptidase A (50 U/mL)^c^
(**31**)	H	**CH**_**2**_ **OH**	8.8 h	9.1 h	9.4 h
(**32**)	CH_3_	**CH_2_OH**	12.4 h	11.6 h	12.6 h
(**33**)	H	H	Stable^c^	80.8 h	39.6 h
(**34**)	CH_3_	H	Stable^c^	47 min	23 min

a 0.05 M phosphate buffer at pH 7.4.

b CPA, carboxypeptidase A in 0.05 M phosphate buffer at pH 7.4.

c No degradation was seen after 24 h.

Bold is to emphasize it is correspondent to hydroxymethyl derivative.

Kahns et al. (1993) also used the *N*-hydroxymethylation prodrug approach to protect the peptide *C*-terminal amide bond against α-chymotrypsin, another type of pancreatic proteolytic enzyme. The main objective was to improve the peptide cell uptake, overcoming the enzymatic barrier. In physiological conditions, *N*-hydroxymethylated compounds were completely stable. These studies were performed in buffer solutions (pH 7.4 at 37°C), containing the enzyme in different concentrations (0.25 mg/ml, 0.50 mg/ml, and 1.0 mg/ml). The CH_2_OH group in compounds (**35**–**36**) maintained the same profile of stability in all the assay conditions (Table 3). The derivatives (**37**–**38**) showed increased linear degradation in higher enzyme concentration.

**TABLE 3:** Half-life (t½) in pH 7.4 and in buffer containing α-chymotrypsin (Kahns, Friis, Bundgaard, 1993).
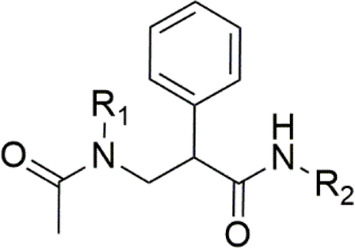

**t½**						
**Buffer with α-chymotrypsin**						
**Compound**	**R1**	**R2**	**Buffer pH 7.4**	**0.25 mg/mL**	**0.50 mg/mL**	**1.0 mg/mL**
(**35**)	**CH**_**2**_ **OH**	H	38 min	33 min	35 min	32 min
(**36**)	**CH**_**2**_ **OH**	CH_2_C_6_H_5_	63 min	65 min	64 min	63 min
(**37**)	H	H	stable	9.6 h	4.9 h	2.3 h
(**38**)	H	CH_2_C_6_H_5_	stable	15.0 h	7.5 h	3.9 h

Phosphate buffer, 0.1 M (pH 7.4). Bold is to emphasize it is correspondent to hydroxymethyl derivative.

5-Fluorouracil (5-FU) (**20**) has been employed for cancer treatment, although it is not available for oral administration. 5-Fluoro-1-(tetrahydro-2-furanyl)-2.4(1*H*,3*H*)-pyrimidinedione (Tegafur – **39**) is a 5-FU prodrug quite widely used as an anticancer agent (Supplementary Schemes 4S, 6S). New prodrugs derived from tegafur were described by Engel and coworkers (2008). Tegafur has a hydroxymethyl metabolite (**40**), and Engel and coworkers assessed the importance of this metabolite to the drug’s activity. It was showed that tegafur (**39**) presented an IC_50_ of 201 μM in sensitive HT-29 human colon cancer cells, whereas the tegafur metabolite (**40**) is more active with IC_50_ of 67 μM in the same cell line, demonstrating the metabolite’s relevance in tegafur’s anticancer action (Engel et al., 2008).

The enediyne core structure (Supplementary Figure 1S) has known antitumor activity due to its reactivity that leads to DNA cleavage. Considering that effect, Tachi and coworkers (2006) designed enediyne prodrugs in order to regulate the reactivity through a stable precursor able to be metabolized *in vivo* to highly reactive enediyne. The enediyne prodrugs containing hydroxymethyl groups showed DNA-cleaving activity. DNA strand scissions observed were about 49 and 41% for compounds (**41**) and (**42**), respectively, at 1.0 mM drug concentration (Tachi et al., 2006).

## Analog Design Effects of Hydroxymethylation on Drug/Bioactive Compounds

Apart from the use of hydroxymethylation in the prodrug design, there are different applications of hydroxymethyl compounds in the analog design for either the optimization of lead compounds or drugs. Several analogs obtained from hydroxymethylation have been described in the search for therapeutic agents to be used in various diseases, such as cancer and viral diseases. Overall, we observe an influence of the hydroxymethyl group over biological activity. In contrast to prodrugs, the hydroxymethyl analogs do not need to be biotransformed into the prototype. They act *per se*, with this moiety.

**FIGURE 2:**
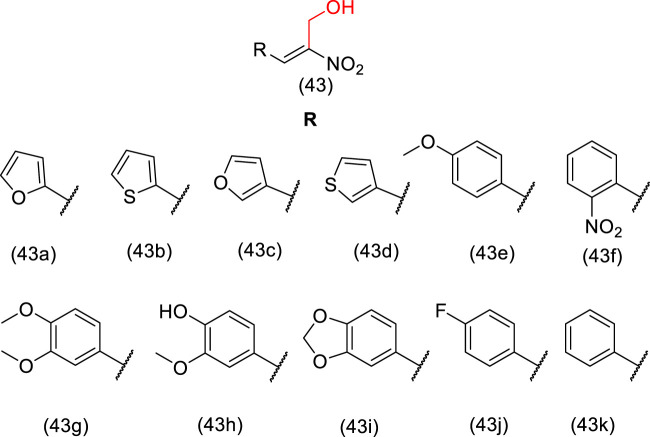
Hydroxymethyl nitroalkene compounds (**43a**-**k**) active in depolymerization of microtubules.

Many anticancer drugs act on microtubules, which are involved in cell division and mitotic processes, such as paclitaxel, vinblastine, and estramustine (Hamel, 1996; Jordan and Wilson, 2004). Hydroxymethyl nitroalkenes derivative (**43**) (Figure 2) showed antiproliferative activity due to depolymerization of microtubules through binding to tubulin, with activity comparable to that of estramustine (Sangrajrang et al., 1998; Nicholson et al., 2002; Mohan et al., 2006). It is worth mentioning that the introduction of the hydroxymethyl group in the nitroalkenes resulted in a reduction in IC_50_ values, which equates to higher activity than nitroalkenes without the hydroxymethyl moiety. Therefore, these analogs (**43a**-**k**) are potential lead compounds that could be useful for anticancer therapy (Mohan et al., 2006). The mechanism of action suggested is the partial involvement in depolymerization of cellular microtubules *in vitro* through tubulin binding to a different site compared to vinblastine and colchicine (Mohan et al., 2006).

Noscapine is a well-known alkaloid isolated in 1817 from *Papaver somniferum* L (opium), which exhibits antitussive activity. Among several potent noscapine derivatives synthesized by Tomar et al. (2019), it was reported that the hydroxymethylation at the *C*-9 position (Supplementary Figure 2S) of the isoquinoline ring (**44**) reduced IC_50_ in U87 glioblastoma cells (4.6 µM) and U251 resistant glioblastoma cells (32.6) compared to noscapine (46.8 μM and 75.4s μM, respectively). The authors attributed this greater activity to the hydroxymethyl group, which may be associated with greater solubility of this compound (**44**) and a better pharmacokinetic profile, allowing superior penetration through the membrane of cancer cell lines.

**FIGURE 3:**
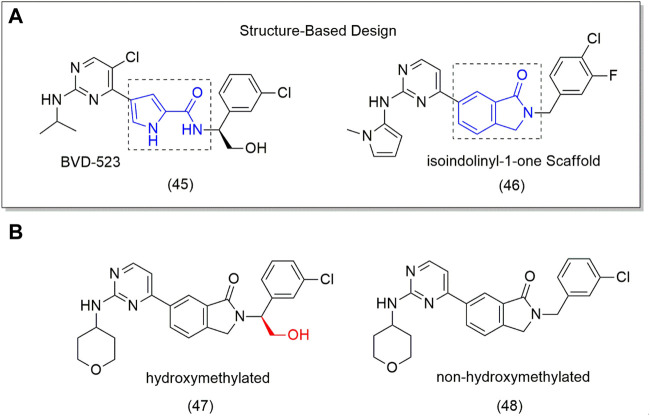
**(A)** Drug Design from 4-pyridone to isoindolinyl-1-one (46). **(B)** ERK1/2 inhibitors (**48**) and the hydroxymethyl compound (**9**).

One of the potential targets for designing new anticancer drugs is the extracellular signal-regulated kinase (ERK), an important protein in the mitogen-activated protein kinase (MAPK) pathway responsible for inducing cell proliferation, differentiation, motility, and survival. Mutation of MAPK (such as MEK and BRAF) pathways is involved in several tumor cells. MEK and BRAF inhibitors are already in clinical use, while ERK inhibitors have failed clinical trials, for example, BVD-523 (**45**) (Figure 3A), an ATP competitive ERK inhibitor. Despite this, the BVD-523 (**45**) molecule was employed as a scaffold for designing orally bioavailable ERK1/2 inhibitors (**46–48**). The results showed that the hydroxymethylated derivative (**47**) showed to be 5-fold more active (IC_50_ = 0.7 nM) on ERK2 and twice on ERK1 than the non-hydroxymethyl analog (**48**) (IC_50_ = 35.6 nM) (Figure 3B); however, none of them was superior to BVD-523 (**45**) (IC_50_ = 0.7 nM), indicating that the hydroxymethyl group is essential to biological activity (Ji et al., 2019). This can be attributed to better interaction with the target through H bonding.

**SCHEME 5:**
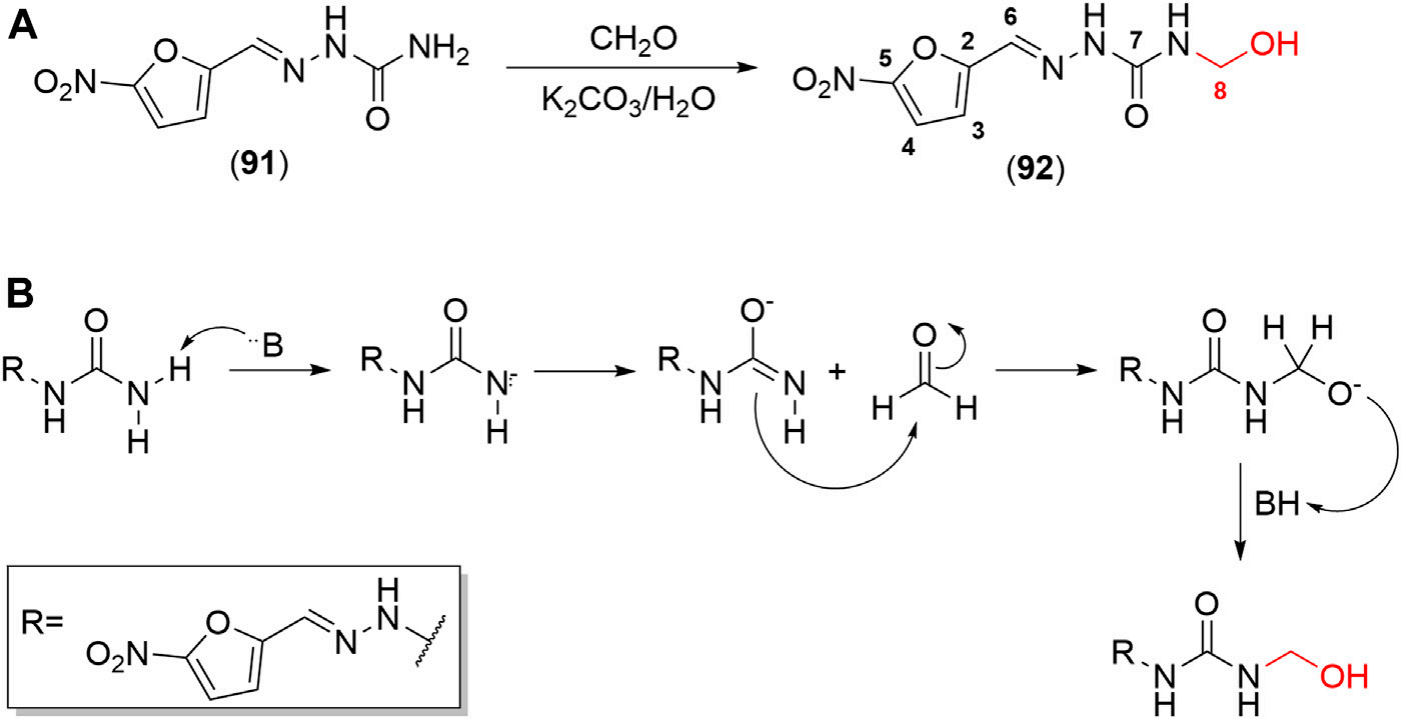
**(A)** Scheme of synthesis of NFOH (**91**). **(B)** Mechanism for the formation of Mannich bases, which has the hydroxymethyl derivative as an intermediary in one of its mechanisms.

Hydroxymethyl compounds were also studied as angiogenesis promoters, which may act in the treatment of diseases involved with lack of blood flow, such as Buerger’s disease or arteriosclerosis. Angiogenesis is a pathway for generating new capillaries from pre-existing vessels, which is important for physiological and pathological processes, such as wound healing, tumor growth, and metastasis (Bobek et al., 2006; Collinson and Donnelly, 2004; O´Toole et al., 2001). Considering this effect, Tsukamoto et al., (2010a) reported the first angiogenesis promoter of low molecular weight, a soluble and stable hydroxymethyl derivative, named 2-chloro-carbocyclic oxetanocin A (COA-Cl - **49**) which induces the phosphorylation and activation of MAPK, responsible for inducing angiogenesis and tube formation (Tsukamoto et al., 2010b; Sakakibara et al., 2017). With the aim of designing more potent COACl analogs (Sakakibara et al., 2015), synthesized dihydroxymethyl compounds conjugated to the cyclobutane ring in positions 2 and 3 or around the functional group in position 2 of the COA-Cl purine skeleton. All compounds exhibited good to moderate angiogenic activity, and the derivatives (**49b**, **49d**, and **49e –** Scheme 5; Supplementary Scheme 7S) were the most active with relative tube areas of 3.43 ± 0.44, 3.32 ± 0.53, and 3.59 ± 0.83, respectively, which were similar to that of COA-Cl (3.91 ± 0.78). The molecular modification did not improve biological activity.

Both vanillin (**50**), a natural compound, and its derivative isovanillin (**51**) have anticancer properties (Supplementary Schemes 5S, 8S). A total of twenty-one isovanillin derivatives were created to evaluate antitumoral activity against diverse cancer cell lines, such as B16F10-Nex2, HL-60, MCF-7, A2058, and HeLa. The most promising compounds have an alkyl chain ranging from 9 to 15 carbons at C_3_, where the hydrophobic chain increases the cell permeability and consequently the cytotoxic activity. However, reducing the aldehyde group (**52**) to a hydroxymethyl derivative (**53**) (Carvalho et al., 2019) revealed activity improvement in all cells tested. Based on the reactivity of the structure, the aldehyde derivatives were more susceptible to nucleophiles. Despite this, however, the hydroxymethylated derivatives showed better results, demonstrating the versatility of the hydroxymethylated group, probably due to other factors such as increased solubility.

**FIGURE 4:**
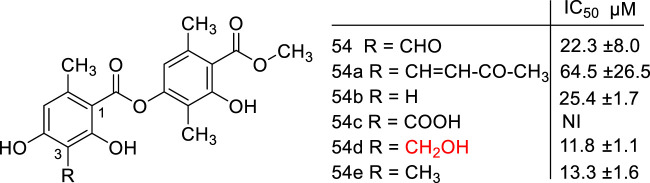
Atranorin (**54**) and its derivatives (**54a** – **e**).

The same profile of results occurred with atranorin (**54**), a metabolite isolated from the lichen *Stereocaulon evolutum* that exhibited anti-HCV activity. The molecular modifications in the C_3_ aldehyde group obtained new antiviral derivatives (**54a**-**e**) (Figure 4). The hydroxymethyl analog **(54d**) displayed the best activity (IC_50_ = 11.8 µM), which was higher than the aldehyde (**54**, IC_50_ = 22.3 μM) and methylated derivatives (**54e**, IC_50_ = 13.3 μM) (Vu et al., 2015).

**FIGURE 5:**
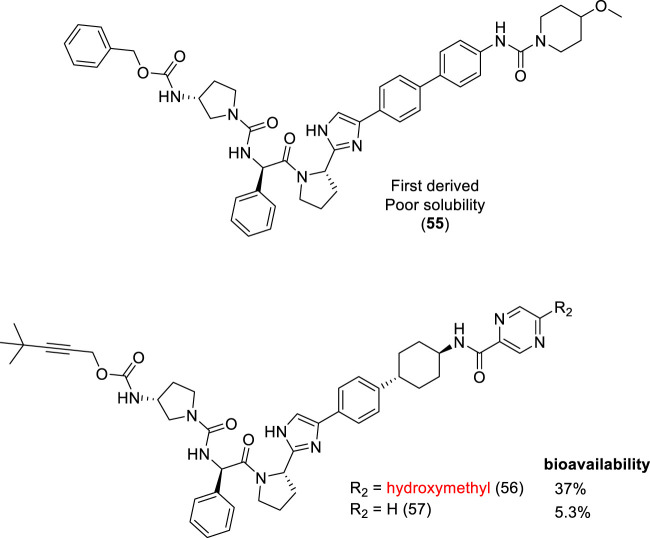
Hydroxymethyl derivatives as HCV NS5A inhibitors (**56**).

Daclatasvir is a nonstructural 5A protein (NS5A) inhibitor, involved in hepatitis C virus (HCV) replication. Daclatasvir was employed as a scaffold for designing new inhibitors. The first analog (55 – Figure 5) exhibited inadequate properties, and it was classified into the Biopharmaceutics Classification System (BCS) (Amidon et al., 2014), as class IV due to its poor solubility and permeability. Nakamura and coworkers (2020) developed a series of compounds based on the first poorly soluble derivative (55). Among the several derivatives obtained, the compound containing the hydroxymethyl group at R2 position (**56** – Figure 5) exhibited better solubility and the highest oral bioavailability, with EC_50_ values in the picomolar range, inhibiting all HCV genotype 1a (0.059), 1b (0.013), 2a (<0.01), and 3a (<0.01) replicons and 1a Q30 mutants. This simple molecular modification increased the bioavailability about 7-fold over a non-hydroxymethyl analog (57) (Nakamura et al., 2020).

Hydroxymethylation was also used to improve antiviral derivative developed by Guo and coworkers (2012) who identified a novel class of potent anti-HIV agents, named 3′,4′-*di*-*O*-(*S*)-camphanoyl-(+)-*cis*-khellactone (DCK – **58**) (Supplementary Figure 5S), which was able to inhibit the production of viral HIV-1 double-stranded DNA from a single-stranded DNA intermediate, a new mechanism of action. DCK was chosen as a candidate for preclinical assays due to its high potency (EC_50_ = 0.0015 μM) and easy synthesis. 3-Hydroxymethyl-4-methyl-DCK (HMDCK - **59**) that was obtained which showed high potency (EC_50_ = 0.004 μM) and moderate bioavailability in mice. Despite improvement of these properties, it did not show enough metabolic stability (Guo et al., 2012). This example shows that success is not guaranteed with a hydroxymethylated compound, as it is a metabolically unstable group.

Borah et al. (2015) reaffirm that the hydroxymethyl groups’ pharmacophore is to bind protein kinase C (PKC), a family involved in several cell signalizations, such as transduction, proliferation, apoptosis, and metabolism. This evidence is based on structure–activity relationship (SAR) studies, which demonstrated that the hydroxymethyl and carbonyl functional groups of diacylglycerols (**60**, DAGs -), DAG-lactones (**61**), and phorbol esters (**62**) play an important role in their interactions with the backbone amide protons and carbonyl of PKC-C1 domains (Wender et al., 1988). Therefore, it comes as no surprise that the phorbol esters exhibited more than a 1000-fold stronger binding affinity for a C1 domain than structurally simple and flexible DAGs. Borah and colleagues designed (S)-γ-hydroxymethyl-γ-butyrolactone (**63**–**64**, HGL) analogs with the hydroxyl and carbonyl groups within the same molecule and a hydrophobic chain for membrane interaction. The compounds derived from the molecule **(64)** showed more than 9- and 15-fold stronger binding affinity for the PKC-C1 subdomain than DAGs, respectively (Supplementary Figure 6S).

Kurkinorin is the first centrally acting, non-nitrogenous μ-opioid receptor salvinorin-derived agonist, acting on the pathway for β-arrestin-2 recruitment. With the objective of designing compounds without the adverse effects common to current opioids, such as tolerance, dependence, and respiratory depression, replacements at the 2, 3, and 4 positions of aromatic and bioisoster rings were performed. The most promising analog (**65**) (Supplementary Figure 7S) was the hydroxymethylated one at position 4 of the ring. *In vitro* assays showed potent activity of this hydroxymethyl-derived compound (EC_50_ value of 0.03 ± 0.01 nM) and selectivity at the μ-opioid receptor over the kappa-opioid receptor with a tendency toward G-protein activation. In addition, the hydroxymethyl compound was about 100-fold more active than morphine (EC_50_ 5.04 ± 3 nM) and 5-fold more potent than fentanyl (EC_50_ 0.26 ± 1 nM), exhibiting analgesic effects without significant tolerance effects in *in vivo* assays (Crowley et al., 2020). It is worth noting that the hydroxymethyl group displays a role in the increased potency, through interaction by hydrogen bonding with the receptor.

Hydroxymethyl bioisoster of phenolic GluN2B-selective *N*-methyl-d-aspartate (NMDA) receptor antagonists were designed for the treatment of several neurological disorders, including neurodegenerative diseases. The NMDA receptor is involved in the development of neurodegenerative diseases, such as Alzheimer’s disease, Huntington’s disease, and Parkinson’s disease. This receptor is composed of two subunits of GluN1 and two of GluN2B. Considering this (Temme et al., 2018), synthesized compounds replace the phenol with the hydroxymethyl group because phenol undergoes fast hepatic metabolism by glucuronidation and sulfation. The derivative (**66**) showed a promising GluN2B affinity (Ki = 101 nM) (Supplementary Figure 8S). In addition, it exhibited high selectivity, without interacting with the phencyclidine (PCP) binding site of NMDA, which is responsible for the side effects from opioids. It is believed that the hydroxymethyl moiety can change the conformation of the channel through hydrogen bonding, keeping it closed.

5-Hydroxymethyl-2-furfural (5-HMF, **67**) (Supplementary Schemes 5S, 9S) is a hydroxymethyl compound and major product of carbohydrate metabolism from the reduction of sugars and amino acids (Murkovic and Bornik, 2007) and is present in honey, coffee, fruits, and flavoring agents (Van-Gorsel et al., 1992). Abdulmalik et al. (2005) reported that 5-HMF (**67**) forms a stable Schiff base adduct with intracellular HbS (S- hemoglobin) when incubated with sickle cells, shifting the oxygen equilibrium curve toward the left and inhibiting sickling in these cells. These findings suggest that 5-HMF (**67**) may assist in the treatment of sickle cell disease. In another study conducted by Ya et al. (2012), 5-MHF (**67**) was able to prolong the survival of mice with permanent forebrain ischemia and protect against hypoxia.

In the drug design, the use of methyl groups is quite common due to their versatility, steric effect, hydrophobicity, and small size (Barreiro et al., 2011), compared to other alkyl groups, although not when compared to hydrogen. Therefore, methyl can compose linear and branched chains and aromatic rings, and it can be used as a bioisoster of several groups. The methyl group can undergo biotransformation and be converted to a hydroxymethyl derivative. This metabolite is further oxidized by CYP450 enzymes, forming metabolites which can be more active than the parent drug. Considering this effect, Δ9-tetrahydrocannabinol (**68**) is a psychoactive constituent from *Cannabis sativa* L. THC has a great homeostatic influence on the central nervous system (CNS). The carbon C11 is oxidized to a hydroxymethyl metabolite, 11-hydroxy-Δ-9-tetrahydrocannabinol (**69**), responsible for the most pharmacological effects (Supplementary Schemes 6S, 9S) (Sharma et al., 2012; Mechoulam et al., 2014; El-Haj and Ahmed, 2020).

## Examples of Compounds Containing a Hydroxymethyl Group in Therapeutics

It is worth noting that some of the examples from natural sources herein presented are hydroxymethylated *per se* and not submitted to a hydroxymethylation reaction. However, many articles stressed that the evidence points to this group playing a significant role in their activity. As observed in other examples, molecular modification, as hydroxymethylation, can be based on structures coming from natural sources.

In hypertension research, the renin-angiotensin system has a fundamental role in the control of cardiovascular homeostasis and electrolyte balance. The angiotensin type 1 (AT1) receptor controls angiotensin II, being responsible for activities in homeostatic control, such as vasoconstriction, aldosterone release, sympathetic nervous system action, and cell growth. The search for AT1 receptor antagonists has been continuous for hypertension treatment (Dasgupta, and Zhang, 2011). In this class of drugs, the Dupont group introduced a hydroxymethyl group at position 5, resulting in the losartan structure (**70)** (Supplementary Figure 10S), which allowed hydrogen bonds with the receptor (Kohara et al., 1996; Yanagisawa et al., 1996).

Another target in cancer treatment is DNA methylation, which is responsible for changing the gene regulation (Zemach et al., 2010; Bird, 2002; He et al., 2011; Law and Jacobsen, 2010). In this context, azacitidine (**71**) and its derivative 5-aza-2′-deoxycytidine (decitabine - **72**) (Supplementary Figure 11S) are synthetic drugs approved by the FDA for treating myelodysplastic syndromes (Glover et al., 1987). These therapeutic agents (**71**–**72**) are hydroxymethyl-based drugs and DNA methyltransferase inhibitors, which induce DNA demethylation (Selvara jet al., 2010; Bracht et al., 2012; Yan et al., 2014). The authors observed that the hydroxymethyl moiety in these drugs is essential to phosphorylation reactions and consequently needs to be activated for biological activity. The search for epigenetic drugs, such as azacitidine and decitabine in low concentrations may prevent the formation and growth of cancer and progenitor cells, is ongoing. These types of drugs can also reduce cancer recurrence. The bioactive agents mentioned previously (**71**–**72**) show a promising future as anticancer epigenetic drugs. Additionally, they may be more effective, since they eliminate progenitor cancer cells (Sarkar et al., 2013; Yan et al., 2014).

Kojic acid (**73**; 5-hydroxy-2-(hydroxymethyl)-4*H*-pyran-4-one – Supplementary Figure 12S) is a natural hydroxymethyl compound produced by several species of fungi, mainly *Aspergillus oryzae* *.* Kojic acid and *p*-hydroxybenzyl alcohol are tyrosinase inhibitors used worldwide in cosmetic formulation for skin whitening and to treat melasma (Chang, 2009). In 2015, Premi et al., (2015) and coworkers reported that exposure to UVA (ultraviolet A) for more than 3 h can produce dark cyclobutane pyrimidine dimers (CPD) in melanocytes, which are responsible for developing melanomas. Moreover, kojic acid (**73**) at 50 µM suppresses the production of dark CPDs. *p*- Hydroxybenzyl alcohol (**74**), which is synthetic, is able to inhibit melanogenesis at noncytotoxic concentrations, showing specific inhibition on cellular tyrosinase without effect on the *m*RNA tyrosinase expression level. In addition, *p*-hydroxybenzyl alcohol shows anti-inflammatory action, due to its ability to decrease cyclooxygenase activity and prevent the release of pro-inflammatory mediators, such as nitric oxide (NO), besides inhibiting angiogenesis, thus suggesting an anticancer agent (Liu et al., 2007).

The importance of a single hydroxymethyl group in some biological activity can be observed in the effect of dihydroresveratrol glucoside (**75**), isolated from *Camellia oleifera* Abel, and its synthetic derivative xyloside (**76**) (Supplementary Figure 13S). The hydroxymethylated compound (**75**) is about 40-fold more potent than kojic acid as a melanogenesis inhibitor in B16F0 melanoma cells, in contrast to the compound without hydroxymethyl (**76**) that stimulates melanogenesis. Therefore, the hydroxymethyl group turns the resveratrol derivative (**75**) into a potent melanogenesis inhibitor (Oode et al., 2014) and emphasizes once again the importance of the hydroxymethyl group.

Another example of a hydroxymethylated compound from natural sources is gastrodin (**77**; 4-hydroxybenzyl alcohol 4-*O*-β-d-glucopyranoside – Supplementary Schemes 7S, 10S), the major component found in *Gastrodia elata*, a traditional Chinese herbal. Gastrodin is a potent antioxidant and anti-inflammatory agent with other pharmacological properties (Chen and Sheen, 2011; Dai et al., 2011) for the treatment of conditions such as dizziness and seizure that has its effect due to decreased GABA activity (An et al., 2003). In CNS, gastrodin (**77**) is a promising agent to treat Alzheimer’s disease (Zhao et al., 2012) due to its neuroprotective effects caused by attenuation of Aβ deposition in the cortex and hippocampus in brains of transgenic mice (Hu et al., 2014). Moreover, gastrodin decreases neuroinflammation through the suppression of cerebral pro-inflammatory cytokines TNF-α and IL1β, produced by microglial cells (Hu et al., 2014). In addition, it is involved in protecting from cerebral ischemic damage in mice (Peng et al., 2015).

Gastrodin (**77**) has also shown to be an effective inhibitor of cardiac hypertrophy and is able to preserve the cardiac function in mice, preventing hypertrophy by blocking ERK1/2 signaling pathways (Shu et al., 2012). Huang et al. (2015) reported that gastrodin inhibits adipogenesis and osteoclastogenesis by decreasing ROS (reactive oxygen species) and osteoclast-specific markers, partially reversing *in vivo* the ovariectomy effects in BALB/c female mice. Therefore, gastrodin (**77**) is a potential anti-osteoporosis herb-derived agent. In its metabolism, gastrodin (**77**) is biotransformed into hydroxymethyl glucoside (**78**) and *p*-hydroxybenzyl alcohol (**74**) (Supplementary Schemes 10S, 12S) (Jia et al., 2014).

**FIGURE 6:**
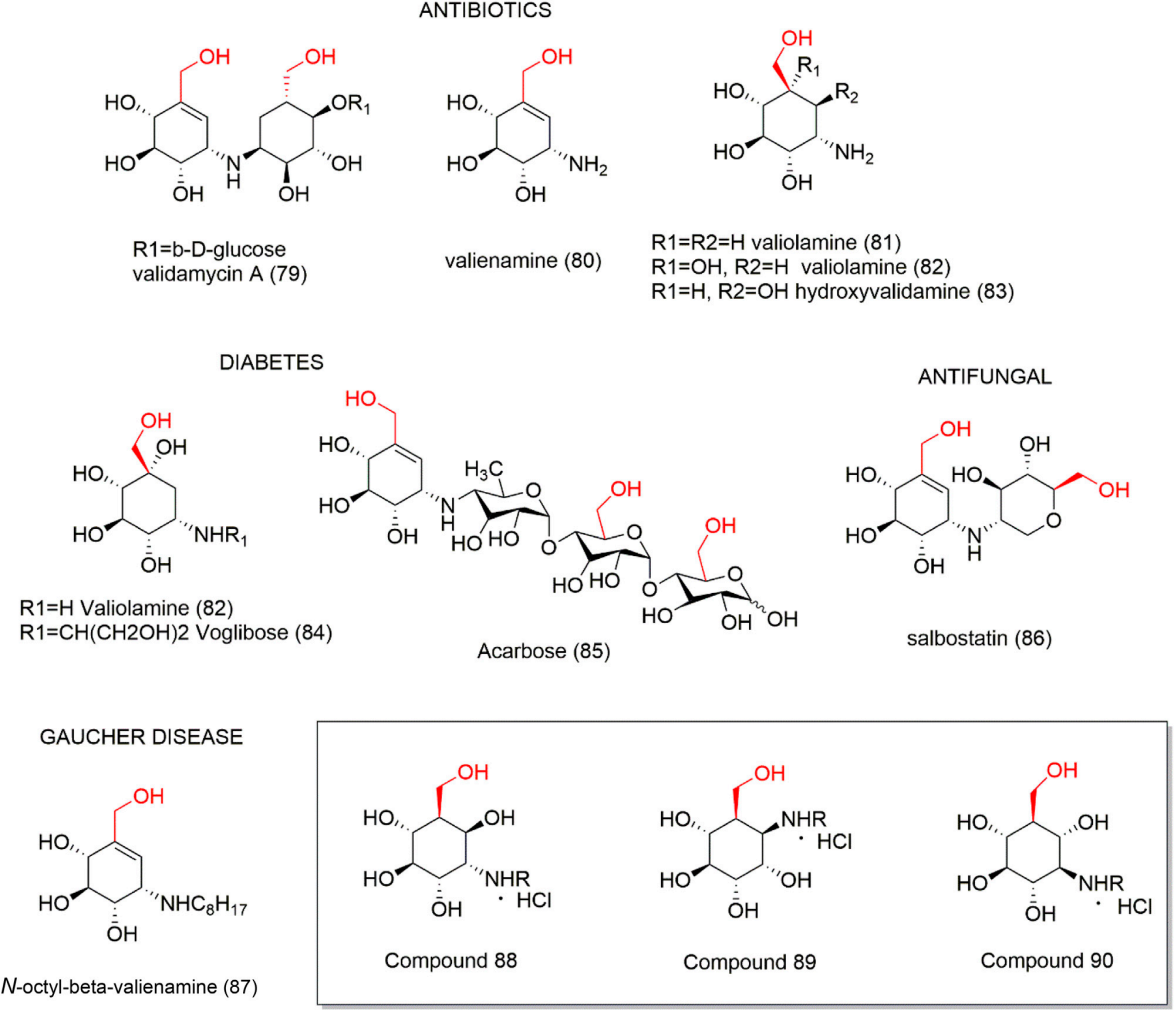
Aminocyclitols containing hydroxymethyl substituent (**79**–**90**).

Aminocyclitols are amino polyhydroxy cycloalkanes able to mimic natural carbohydrates. The amino group modulates biological activity and its replacement by oxygen in the endocyclic ring ensures stability in water. Several aminocyclitol derivatives (**79–87**) have been obtained over the last decades, including compounds with a hydroxymethyl substituent with therapeutic interests (Figure 6), such as antibiotics from the validamycin family (**79**–**83**) and salbostatin (**86**), anti-diabetic agents valiolamine (**82**), voglibose (**84**), and acarbose (**85**), and anti-Gaucher disease agent *N*-octyl-β-valienamine (**87**) (Horii et al., 1974; Kameda et al., 1975; Chen et al., 2003). In this context, the drug acarbose (**85**) is a glucosidase inhibitor and promotes slower carbohydrate digestion, avoiding the increase of postprandial plasma glucose. Thus, acarbose (**85**) is used to treat type 2 diabetes mellitus, mainly in the elderly, due to its safety and prevention of the occurrence of acute endothelial dysfunction and other authors (Harrison et al., 2014; McCarty and Di Nicolantonio, 2015) evidenced many important effects of this compound. Based on the interest demonstrated in these aminocyclitols, Trapero et al. (2015) synthesized a set of aminocyclitol derivatives from cyclohexane epoxides by a stereo-controlled ring opening with nitrogen nucleophiles. These compounds were assayed as glycosidase and recombinant glucocerebrosidase inhibitors. The authors reported that the configurations of amino and hydroxyl groups demonstrated a significant effect on glycosidase inhibition, as the hydroxymethylaminocyclitol analogs (**88**–**90** - Figure 6) were shown to be the highest active compounds as lysosomal β-glucosidase inhibitors.

## Hydroxymethylnitrofurazone: A History of Well-Succeeded Hydroxymethylation

Hydroxymethylnitrofurazone (NFOH - **91**) was first reported by Chung and coworkers (2003), as an intermediate of the Mannich reaction, in one of the mechanisms (Bundgaard and Johansen, 1980a) used to synthesize mutual prodrugs with primaquine (PQ – **92**) and nitrofurazone (NF – **93**) that are potentially active drugs used in Chagas disease. Currently, PQ (**92**) is an antimalarial drug and its antichagasic activity (Kondrashin et al., 2014) is due to active metabolites able to cause oxidative stress in the parasite, leading to *T. cruzi* death (Augusto et al., 1986). However, PQ (**92**) activity was not sustainable, probably due to the action of trypanothione reductase, a key enzyme in the parasite defense mechanism, acting as an antioxidant (Cazzulo et al., 2001). On the other hand, NF (**93**) is an antibacterial agent, therapeutically used in burns, and is a trypanothione reductase inhibitor (Henderson et al., 1988). In this context, mutual prodrugs containing PQ (**91**) and NF (**92**) could have synergic activity due to dual mechanisms of action. With the purpose of accomplishing a selective *in vivo* biotransformation, the dipeptides Lys-Arg, Phe-Ala, and Phe-Arg (**94**) were employed as spacers and targeting groups for cruzipain, a cysteine-protease specific to *T. cruzi,* and responsible for preferentially cleaving these dipeptides (Cazzulo et al., 1990). Thus, upon enzyme recognition, PQ (**92**) and NF (**93**) should be delivered selectively inside the parasite (Scheme 3). These prodrugs inhibited the growth of the epimastigote, amastigote, and trypomastigote forms of *T. cruzi*. The most active compound was an intermediary of mutual prodrug synthesis, the simple molecule NFOH (**91**) (Chung et al., 2003). This fact demonstrated that the inclusion of a hydroxymethyl group in nitrofurazone brought several benefits. The results of studies performed with NFOH (**91**) are summarized in Scheme 7.

### Synthesis and Physicochemical Properties

NFOH (**91**) is an easily synthesized compound from NF and formaldehyde in potassium carbonate at room temperature (Scheme 5A), following the mechanism of Mannich base formation (Scheme 5B) (Chung et al., 2003). In addition to the simplicity of synthesis, NFOH (**91**) is a low-cost compound, and the reagents are easily available, which is outstanding, considering its possible application in a disease prevalent in neglected populations. A factorial experiment was carried out in 2007 to increase reaction yield to 87% of NFOH (**91**) in 7 h (Trossini et al., 2010b). According to the structural elucidation by crystallography-based X-ray, NFOH (**91**) is essentially planar, except for the hydroxyl group (1.106 (7) A˚ deviation) and the oxygen atoms of the nitro group, which are slightly deviated from the planar structure (Doriguetto et al., 2005).

La-Scalea et al. (2005) showed that hydroxymethylation of NF (**93**), which did not change its voltammetric behavior suggesting the formation of nitro anions in NFOH (**91**), is not related to the mechanism of action/toxicity. The inclusion of the hydroxymethyl group led to druggability of NFOH (**91**) by oral route, resulting in higher water solubility than NF (**93),** which was further increased through cyclodextrin (Grillo et al., 2007; Grillo et al., 2008a; Grillo et al., 2008b; Grillo et al., 2010) or nanoparticle formulations (Monteiro et al., 2014). It is worth mentioning that NFOH (**91**) follows Lipinski’s rule of five, exhibiting a molecular weight of 228 g/mol, cLog*P* value of -1.05, three group H bond donors, and three group H bond acceptors.

### Biological Activity Against Chagas Disease

NFOH (**93**) was tested in an *in vitro* assay, using cell culture of rhesus monkey renal epithelial cells (*Macaca mullata*) infected with *T. cruzi* strains to evaluate the growth of trypomastigote and amastigote forms. NFOH (**91**) showed higher activity than benznidazole (BNZ, reference drug), including no recrudescence of *T. cruzi* which was observed with BZN (Chung et al., 2003).

The biggest challenge of *T. cruzi* infection is the chronic phase, since there are no effective drugs for this phase. Davies and colleagues (2010) evaluated the activity of NFOH (**91**), in a subchronic mice model, using Tulahuen strains of *T. cruzi* with NFOH (**91**), NF (**93**), or BNZ for 60 days. Following 180 days of the infection, NFOH (**91**) (150 mg/kg) showed trypanocidal activity similar to BNZ (60 mg/kg) with the lowest mortality rate of only 16%, while NF (**93**) had 75% of mortality and BZN, 33%. From 16% mortality due to NFOH (**91**) treatment, 10% was due to the infection, and after 180 days no parasites were found (PCR tests). Ekins et al. (2015) described the phenotypic high throughput screening (HTS) of over 300,000 molecules using the NIH-Molecular Libraries program. Thus, only five compounds presented promising anti-*T. cruzi in vitro* screening, among them NFOH (**91**), which displayed EC_50_ of 0.775 µM. Moreover, the trypanocidal activity against bioluminescent *T. cruzi* Brazil luc strains in mice models demonstrated 78.5% parasitemia elimination after 4 days of NFOH (**91**) treatment (50 mg/kg^−1^, i.p.) during the acute Chagas stage.

Scarim et al. (2018) induced the indeterminate form of Chagas disease in mice with a Y strain of *T. cruzi* and assessed the histopathological data resulting from NFOH (**91**) and BZN (150 mg/kg^−1^) and BZN (60 mg/kg^−1^) treated for 60 days plus 14 days with the immunosuppressor dexamethasone. The disease reactivation was observed only in the nontreated group. However, the group treated with BNZ exhibited amastigote in the heart and liver, and none was found with NFOH (**91**) (Scarim et al., 2018). A study using a bioluminescent strain of *T. cruzi* (TcVI-CL Brener) demonstrated that a NFOH (**91**) dose of 100 mg/kg, administered for only 5 days, showed no reactivation of *T. cruzi* in a chronic model, after two cycles of immunosuppression with cyclosporine (200 mg/kg) and a 50% reactivation after the third cycle, translating into a 50% cure rate. The *in vitro* and *ex vivo* results also showed a tripanostatic effect of NFOH (**91**), suggesting additional studies with more than 5 days of treatment are necessary (Scarim et al., 2021b). These data assays confirm NFOH (**91**) as a promising and safe new chemical entity to novel preclinical and clinical studies against Chagas disease. Table 4 summarizes the animal activity results.

**TABLE 4:** Main results accomplished with NFOH in animal studies (Davis et al., 2010; Ekins et al., 2015; Scarim et al., 2018; Scarim et al., 2021a).

Mice	Stage	*T cruzi* strain	Protocol	NFOH mg/kg	BZN mg/kg	Results	Ref.
*Swiss*	Subchronic	Tulahuen	60 days drug treatment	150 (v.o)	60 (v.o)	Mortality: BZN (33.33%); NF(75%)	Davis et al. .(2010)
						NFOH (16%) after 180 days, PCR was negative (NFOH and BZN)	
Balb/c	Acute	*T. cruzi* Brazil Luc	4 days NFOH	50 (i.p.)		Decreased 78.5% parasitemia	Ekins et al. (2015)
Balb/c	Underterminate	Y strain	60 days NFOH +14 days dex^1^	150 (v.o)	60 (v.o)	BZN: presence of amastigotes in the heart and liver; absence with NFOH.	Scarim et al. (2018)
Balb/c	Chronic	*T. cruzi* CL-Luc	5 days NFOH drug treatment +	100 (v.o)	100 (v.o)	NFOH: no reactivation of *T. cruzi* after two cycles	Scarim, et al. (2021b)
			3 cycles^2^ (cyclophosphamide)			50% animals cured after three cycles; *in vitro* and *ex vivo* assays suggest trypanostatic activity	
						More days of treatment needed	

1 14 consecutive days of dexamethasone.

2 one cycle each 3 days with cyclophosphamide.

BZN: beznidazole, the reference drug.

### Studies on the NFOH (91) Mechanism of Action

Barbosa et al. (2007) assessed, *in vitro*, the capability of NF (**93**) and NFOH (**91**) to act on *m*RNA processing during *trans*-splicing reactions. The findings showed that NFOH (**91**) interferes in RNA processing by a partial inhibition of the *trans*-splicing reaction, in Y and NCS *T. cruzi* strains. Also, NFOH (**91**) showed higher cruzain inhibition from *T. cruzi* (IC_50_ = 10.55 ± 0.82 μM) than NF (**93**) (IC_50_ = 22.83 ± 1.2 μM), at the same concentration (10 μM). NFOH (**91**) and its derivatives showed a 60% inhibition of enzymatic activity against 30% inhibition observed with NF (**93**). In addition, the electronic surface map and molecular docking of NFOH (**91**) in cruzain indicate a favorable attack of the Cys-25 of the enzyme on its carbonyl group due to the lower electronic density of the NFOH (**91**) -carbonyl moiety. Considering that NFOH (**91**) can also act by forming nitro radicals, this suggests a dual mechanism of action (Trossini et al., 2010). Further studies are needed to elucidate NFOH (**91**) mechanism of action.

### Toxicity Evaluation

Nitro compounds are known to present higher toxicity; however, there are many examples of nitro-based derivatives in therapy (Chung et al., 2011). The intermediates formed, responsible for cytotoxicity of nitro compounds, are hydroxylamine derivatives and nitro radical anions (Edwards, 1990; Moreno and Docampo, 1985; Viodé et al., 1999). DNA damage caused by nitro compounds depends on several factors, such as the stability of the anion formed and the reactivity of the DNA molecule inducing genotoxicity (Maya et al., 1997). In this context, one of the major concerns with nitro compounds is genotoxicity. NFOH (**91**) was evaluated *in vitro* through a reverse gene mutation assay (Ames test), using *Salmonella typhimurium* strains TA102 and TA98, either in the presence or the absence of the fraction S9 of the rat liver. NFOH (**91**) showed four times lower mutagenicity in both strains tested (with or without metabolic activation) than the parent drug NF (**93**) (Güido et al., 2001). In addition, Chung et al. (2011) assessed the genotoxic potential of NFOH (**91**) and NF (**93**), using a micronucleus model in mice blood, employed to analyze the capacity of substances to induce chromosomal injury in cells in division. The findings showed that NFOH (**91**) presented a profile similar to water, while BNZ and NF (**93**) induced damage to the micronucleus.

NFOH (**91**) acute and subacute toxicity tests were performed in mice and rats treated with NFOH (**91**) orally (p.o.) or intraperitoneally (i.p.), evaluating the delayed death after a single dose at a 14-, 28- and 60-days observation period, and the results showed LD_50_ rats (p.o) > 2000 mg/kg and mice(i.p) 327.9 mg/kg for hydroxymethylated compound against 556.3 mg/kg and 197.1 mg/kg for NF, respectively. This study suggested low toxicity of NFOH (**91**). Also, NFOH (**91**) hepatotoxicity was assessed in *in vitro* experiments with HepG2 cells, and it caused an enhancement in ROS generation and in the oxidative damage to DNA biomarker 8-oxo-29-deoxyguanosine (8-oxo-dG). Despite this, cell viability was not significantly changed by the administration of NFOH (**91**). On the other hand, the cells treated with BNZ showed no increase in this biomarker, but they exhibited a reduction in cell viability, displaying 33% cell death at 100 μM (Davies et al., 2014). The results indicated that NFOH (**91**) (5–100 μM) induces a mild response through oxidative stress, but it does not interfere in cell viability, whereas BNZ is cytotoxic at 100 μM, leading to cell death in 4 h. In *in vivo* assays developed in mice, liver injury was assessed by measuring the increase in glutamate oxaloacetate transaminase (GOT) and glutamate pyruvate transaminase (GPT), and NFOH (**91**) exhibited no significant enhancement of GOT and GPT when compared to normal controls, in short- and long-term treatment. Although, the long-term BNZ treatment induced a 5-fold increase in GPT and GOT, demonstrating hepatic impairment. In the TNF-α and inflammatory infiltrate determination, NFOH (**91**) was shown to be well-tolerated in long-term treatment, and BNZ presented extensive tissue inflammation. Therefore, these findings suggested that treatment with NFOH (**91**) was not hepatotoxic, though BNZ treatment was shown to be hepatotoxic and also to induce chronic inflammation and liver injury.

### Pharmacokinetic Studies

The hydroxymethylation improved pharmacokinetic profile of NF (**93**) (Serafim et al., 2013) was assessed in animal, human plasma, and in rats (Nogueira et al., 2013). The molecular modification increased the volume of distribution (Vd), which was 20-fold higher than that of NF (**93**) in rats and 10% higher than that of in rabbits (Table 5).

**TABLE 5:** Pharmacokinetic parameters determined after a single dose (200 mg/kg) of NF (**93**) and NFOH (**91**) in Wistar rats (Serafim et al., 2013) and in rabbits (Nogueira et al., 2013).

Parameter	Rat		Rabbit	
	**NFOH** (**91**)	**NF** (**93**)	**NFOH** (**91)**	**NF** (**93**)
**kel (h** ^**−1**^ **)**	0.099	0.178	0.2664	1.5
**Elimination half-life (h)**	7.00	3.9	2.60	4.60
**Cmax (µg/mL)**	0.99	2.78	3.68	0.263
**Tmax (h)**	1	4	0.75	1
**AUC 0-t (µg/mL.h)**	5.683	54.49	29,686.2	14,632.2
**AUC 0-∞(ug/mL.h)**	5.986	63.70	32,334.6	50,687.4
**Cl*/F* (L/h.kg)**	33.41	3.140	8.96	5.015
***Vd/F* (L/kg)**	337.5	17.64	33.643	30.623

NF, nitrofurazone administered by gavage; NF*, NF obtained by hydrolysis of administered NFOH; Cmax, maximum concentration; Tmax, maximum time; kel, elimination constant; t1/2, half-life; CL*/F*, clearance/oral bioavailability; *Vd/F*, volume of distribution/oral bioavailability; AUC, area under the curve; MRT, mean residence time.

After identifying the promising profile of NFOH (**91**), the design of new prodrugs (Giarolla et al., 2010; Giarolla et al., 2012; Giarolla et al., 2013), targeted drugs (Santos et al., 2013), bioisosters (Serafim et al., 2014; Serafim et al., 2017; Silva et al., 2016), and molecular hybrids from NFOH (**91**) were performed. In an attempt to improve NFOH (**91**) water solubility even more, some cyclodextrin derivatives were prepared for inclusion (Grillo et al., 2007; Grillo et al., 2008a; Grillo et al., 2008b; Grillo et al., 2010). In addition, a patent of polymeric nanoparticles with NFOH (**91**) was sought (Monteiro et al., 2014) with the objective of increasing its solubility. This preparation was shown to be active in *Leishmania*.

Finally, in 2018, the NFOH (**91**) findings sparked the interest of Roche, a pharmaceutical company, and our research group participated in a technology acceleration program, named (ASTRo) Applied Science Trail Roche, and the findings were presented to some pharmaceutical industries and also to a consultant of DNDi.

## Concluding Remarks

The prodrug design is a process that deserves more interest particularly in the area of drug solubility where there is potential to enhance the pharmaceutical and pharmacokinetic properties of drugs and bioactive compounds.

In this article, the topic of hydroxymethylation was highlighted within the context of the prodrug design to accomplish higher water solubility and bioavailability for drugs and bioactive compounds under research. Moreover, the hydroxymethyl derivatives were shown to be interesting intermediates to the synthesis of more soluble as well as more bioavailable acyloxymethyl derivatives. Notwithstanding, this topic must be further explored for the prodrug design and for the development of analogs, which do not need to be biotransformed, and can impart better properties mainly related to pharmaceutics and pharmacokinetics as well as improving the interactions of the compounds with their target. The experience of our group with hydroxymethylation in the prodrug design was in part serendipitous, as we isolated hydroxymethylnitrofurazone (NFOH) (**91**) from a Mannich base synthesis. This intermediary was recovered from an unusual mechanism for this kind of reaction but fortunately proved to be the most active compound in the series designed, as was described in this article. Its remarkable activity in *T. cruzi in vitro* and mainly *in vivo* studies, and the properties of this prodrug raised our interest in designing other derivatives to optimize its features even more. We hope to increase the interest on NFOH (**91**) from pharmaceutical industries, since it has some important advantages aside from the properties described in this article, such as its synthesis, which is low cost. It is worth mentioning that NFOH (**91)** is outstanding as a lead and can be a drug candidate for an extremely neglected disease such as Chagas disease.

We also hope that the examples presented herein within many classes of compounds can evoke the interest of researchers in the area of Medicinal Chemistry.
