# Supplementary material for: Drug/Lead Compound Hydroxymethylation as a Simple Approach to Enhance Pharmacodynamic and Pharmacokinetic Properties
Source: Front Chem. 2022 Feb 14;9:734983. doi: 10.3389/fchem.2021.734983 (PMC8883432; doi:10.3389/fchem.2021.734983)
Supplement: Supplementary file 1 [file DataSheet1.docx]

***Supplementary material***


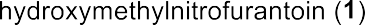

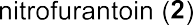


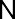

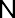

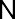

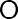

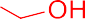

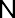

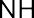

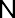

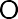


**SCHEME 1S**. The prodrug hydroxymethylnitrofurantoin (**1**) and parent drug nitrofurantoin (**2**).





**SCHEME 2S.** Design of 7-(hydroxymethyl)theophylline prodrugs (**3-6)**.

**SCHEME 3S**. Degradation of allopurinol prodrug (Bundgaard, Falch, 1985).


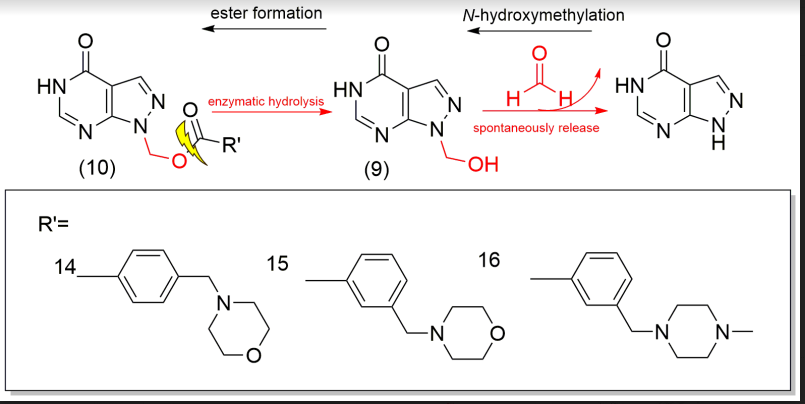


**SCHEME 4S.** Design of *N-*substituted [(3- or 4-(aminomethyl)benzoyloxymethyl]-allopurinol prodrugs (**14-16**).

**SCHEME 5S.** Design of *N*-hydroxymethyl-5FU prodrugs as monocarboxylate transporter 1 (MCT1)-targeting (**21-23**); i 37% formalin, 55 °C, 4h; ii *N,N,N’,N’*-tetramethyluronium hexafluorophosphate (HATU,99%), TEA (trimethylamine), ice bath 1h, then go to room temperature for 5 h.

**SCHEME 6S** Tegafur (**39**) and its hydroxymethyl metabolite (**40**).





**FIGURE 1S**. Enediyne prodrugs with hydroxymethyl groups (**41**-**42**).

**FIGURE 2S.** Hydroxymethyl analogue (**44**) of noscapine.


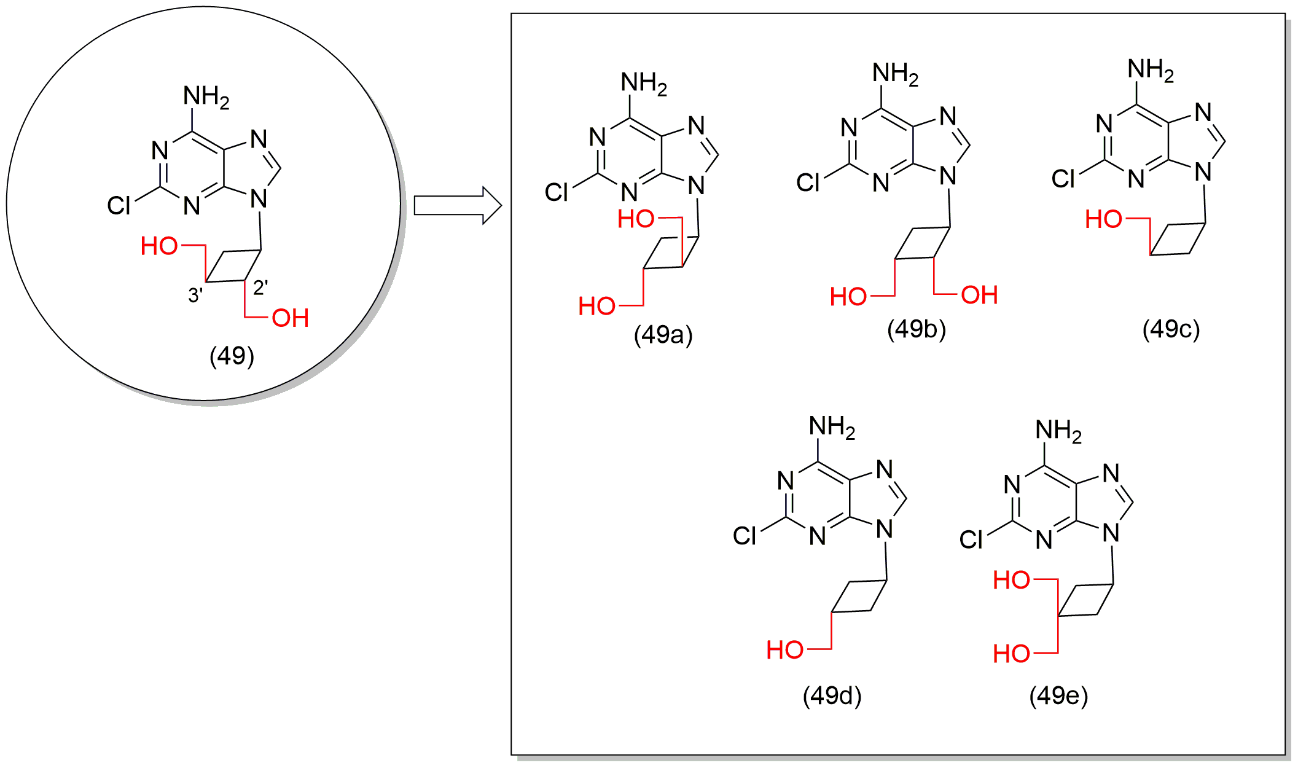


**Scheme 7S.** Hydroxymethyl derivatives (**49a, 49b**, **49c,** **49d**, and **49e**), as potential angiogenesis promoters.

**SCHEME 8S.** Isovanillin (**51**), its aldehyde derivative (**52**), and its hydroxymethyl analogue (**53**). The values of IC_50_ in B16F10-Nex2 were presented for (**51**) and (**53**) respectively (n=9).

**FIGURE 5S**. DCK (**58**) and its hydroxymethyl derivative (**59**).

**FIGURE 6S**. Hydroxymethyl and carbonyl derivatives of diacylglycerols (**60**), DAG-lactones(**63, 64**) and phorbol esters (**63**) able to interact with PKC–C1 domains.

**FIGURE 7S**. Hydroxymethyl derivative kurkinorin (**66**).





**FIGURE 8S.** Hydroxymethyl bioisoster of phenolic GluN2B-selective *N*- methyl-D-aspartate (**66**).

**FIGURE 9S**. 5-hydroxymethyl-2-furfural (5HMF ) (**67**).

**SCHEME 9S**. ∆^9^-THC (**68**) and its hydroxymethyl metabolite (**69**).

**FIGURE 10S**. Losartan (**70**).

**FIGURE 11S.** Azacitidine (**71**) and decitabine drugs (**72**).

**FIGURE 12S.** Tyrosinase inhibitors kojic acid (**73**) and *p*-hydroxybenzyl alcohol (**74**).

**FIGURE 13S.** Structure and activity of dihydroresveratrol glucoside (**75**) and dihydroresveratrol xyloside (**76**).

**SCHEME 10S**. Gastrodin (**77**) from herbs of traditional Chinese medicine.
